# Delayed presentation of an isolated gallbladder rupture following blunt abdominal trauma: a case report

**DOI:** 10.1186/1752-1947-1-52

**Published:** 2007-07-16

**Authors:** Jonathan Bainbridge, Hossam Shaaban, Nick Kenefick, Christopher P Armstrong

**Affiliations:** 1 Department of Surgery, North Bristol NHS Trust, Bristol, UK; 2 Clinical Fellow Upper GI Surgery, Southmead Hospital, Bristol, BS10 5NB, UK

## Abstract

**Background:**

Blunt injuries to the gallbladder occur rarely, and the incidence of isolated damage to the gallbladder is even smaller. We report a case of delayed presentation of isolated rupture of the gallbladder following blunt trauma to the abdomen.

**Case presentation:**

A 65 year old lady presented through the Emergency Department with a 1 week history of blunt trauma to her abdomen. She complained of continued epigastric pain which radiated through to her back and right upper quadrant. On presentation, the patient had a low grade temperature, hypotension and mild tachycardia. Abdominal examination revealed right upper quadrant tenderness with no localised peritonism. C-reactive protein was 451. An abdominal CT showed a moderate amount of ascitic fluid in the perihepatic space. The patient underwent a laparotomy, which revealed a ruptured gallbladder with free bile. There was no evidence of any associated injuries to the surrounding organs. Partial cholecystectomy was done in view of the friable nature of the gallbladder. Post operatively, a persistent bile leak was managed successfully with endoscopic sphincterotomy and stenting.

**Conclusion:**

Rupture of the gallbladder due to blunt injuries to the abdomen occurs from time to time and may constitute a diagnostic challenge especially with delayed presentation. Partial cholecystectomy is a safe option in cases where friability of the wall renders formal cholecystectomy inadvisable. Endoscopic sphincterotomy and stenting is a safe and effective treatment for persistent post operative bile leaks.

## Background

Blunt injuries to the gallbladder occur rarely, and the incidence of isolated damage to the gallbladder is even smaller [1-3]. The delay in presentation of the injury is not unusual. Significant morbidity or even mortality can result from delay in diagnosis, which can easily occur due to both rarity of the condition and low amplitude of symptoms. It is very important to bear in mind the possibility of such injury when confronted with a case of upper abdominal pain following blunt abdominal trauma. We report a case of delayed presentation of isolated rupture of the gallbladder following blunt trauma to the abdomen. A literature review on this subject is also provided.

## Case presentation

A 65 year old lady presented through the Emergency Department with a 1 week history of abdominal pain after being knocked down by a horse she was holding, which resulted in the patient falling onto a stony path and hitting the right side of her abdomen. She complained of continued epigastric pain following the incident, which radiated through to her back and right upper quadrant.

On examination the patient had a low grade temperature (37.5°C) and was hypotensive at 96/61 mmHg, with a pulse rate of 96. Abdominal examination revealed right upper quadrant tenderness with no localised peritonism. Bloods showed a normal full blood count, lipase and liver function but did however reveal a C-reactive protein of 451. Chest and abdominal radiographs were normal with no signs of free air. An abdominal computed tomogram (CT) was performed which showed a moderate amount of ascitic fluid in the perihepatic space, around the porta hepatis and extending down to the pelvis.

The patient underwent a laparotomy, which revealed a ruptured gallbladder with free bile. There was no evidence of any associated injuries to the surrounding organs. Due to the friable nature of the gallbladder and associated inflammation cholecystectomy would have been extremely difficult. Therefore the decision was made to perform a partial cholecystectomy, below the level of the tear, and drainage.

Unfortunately the drain came out unintentionally 3 days after the operation having drained almost 200 mls of bile in that period. There was continued leakage of bile through the drain site increasing in rate to approximately 300 mls per day for the next 48 hours. An abdominal ultrasound at the time showed a continued fluid collection in the pelvis.

Due to the continued bilious drainage the patient underwent an endoscopic retrograde cholangiopancreatography (ERCP) on day 6 post-op. During this procedure a sphincterotomy was performed and a pig-tail stent inserted to allow drainage of the gallbladder. Following this intervention the patient's post-op course was unremarkable apart from a small wound infection. She was eventually discharged home 26 days after admission and the stent was removed 2 months later.

## Discussion

Blunt injuries to the gallbladder occur rarely, ranging from 1.9%, as reported by Penn [4], to 2.1% in the series of patients examined by Soderstrom et al [5]. The incidence of isolated damage to the gallbladder is even smaller, as shown in Soderstrom's review whereby only 5 out 30 cases of gall bladder injuries were isolated. This was also demonstrated by Wiener et al [6], showing that only half of the cases of gallbladder injury were in isolation.

The majority of gallbladder injuries occur following motor vehicle incidents [5-7], significant falls and direct blows in sport e.g. soccer [8], wrestling [9] and rugby [10]. Although there has been an isolated case of injury secondary to a bull head-butting a patients' abdomen [6], there are no identifiable cases of damage occurring with this mechanism of injury. It should also be noted that the patient had eaten in the period preceding the trauma, and therefore the gallbladder was not enlarged in its fasted state. Both the degree of trauma and the absence of any collateral damage make this a unique case to report.

The delay in presentation of the injury is not unusual. Damage to a non-infected gallbladder can cause leakage of sterile bile into the abdomen. This in itself does not present acutely and such injuries can take up to six weeks to become apparent [6,11]. The majority of these cases will be diagnosed peri-operatively, as with our case, although a few cases have demonstrated gallbladder damage using pre-operative computerised tomography [10].

The recommended treatment of gallbladder rupture and major tears is cholecystectomy [5,6,11]. In this case the delayed nature of the presentation resulted in an extremely friable gallbladder, which was not amenable to a total cholecystectomy. This resulted in a partial cholecystectomy being performed, also being a recognised treatment option in such cases. Laparoscopic cholecystectomy is advocated to be a safe and effective procedure in the diagnosis and management of traumatic gall bladder rupture [1]. In our case, however, due to uncertainty of the diagnosis, an exploratory laparotomy was elected as the safest option.

Endoscopic procedures such as sphincterotomy and temporary biliary stenting are well known for their safety and efficacy in the management of persistent biliary leakage post hepatobiliary surgery [12,13]. These procedures act by lowering the pressure at the sphincter of Oddi. This encourages preferential drainage of bile to the duodenum rather than leaking into the peritoneal cavity.

## Conclusion

Rupture of the gallbladder due to blunt injuries to the abdomen occurs from time to time and may constitute a diagnostic challenge especially with delayed presentation. Partial cholecystectomy is a safe option in cases where inflammation and friability of the wall render formal cholecystectomy inadvisable. Endoscopic sphincterotomy and stenting is a safe and effective treatment for persistent post operative bile leaks.

## Competing interests

The author(s) declare that they have no competing interests.

## Authors' contributions

JB is the principal author of the paper, HS participated in collecting data and wrote the endoscopy part of the discussion, NK revised and edited the whole document; CA supervised the project and undertook the final revision before submission. All authors read and approved the final manuscript.
